# Conservation of Expression and Sequence of Metabolic Genes Is Reflected by Activity Across Metabolic States

**DOI:** 10.1371/journal.pcbi.0020106

**Published:** 2006-08-18

**Authors:** Yonatan Bilu, Tomer Shlomi, Naama Barkai, Eytan Ruppin

**Affiliations:** 1 Department of Molecular Genetics, Weizmann Institute of Science, Rehovot, Israel; 2 School of Computer Science, Tel Aviv University, Tel Aviv, Israel; 3 Department of Physics of Complex Systems, Weizmann Institute of Science, Rehovot, Israel; 4 School of Medicine, Tel Aviv University, Tel Aviv, Israel; The University of Tokyo, Japan

## Abstract

Variation in gene expression levels on a genomic scale has been detected among different strains, among closely related species, and within populations of genetically identical cells. What are the driving forces that lead to expression divergence in some genes and conserved expression in others? Here we employ flux balance analysis to address this question for metabolic genes. We consider the genome-scale metabolic model of *Saccharomyces cerevisiae,* and its entire space of optimal and near-optimal flux distributions. We show that this space reveals underlying evolutionary constraints on expression regulation, as well as on the conservation of the underlying gene sequences. Genes that have a high range of optimal flux levels tend to display divergent expression levels among different yeast strains and species. This suggests that gene regulation has diverged in those parts of the metabolic network that are less constrained. In addition, we show that genes that are active in a large fraction of the space of optimal solutions tend to have conserved sequences. This supports the possibility that there is less selective pressure to maintain genes that are relevant for only a small number of metabolic states.

## Synopsis

The regulation of gene product activity allows cells to efficiently cope with various tasks under varying conditions. Given that, one may have expected that striving for efficiency would cause genetically similar cells to have similar regulation. However, in reality, high variations in gene expression levels are detected between different strains and even between genetically identical cells taken from the same culture. What are the driving forces that lead to expression divergence in some genes and conserved expression in others?

To address this question, the authors study the conservation of regulation in yeast metabolism, using a computational model. They find that genes coding for reactions whose flux rates are narrowly constrained by the cellular need to maximize growth rate tend to have strictly conserved regulation and expression. However, when a wide range of flux rates is compatible with high cellular growth rates, the corresponding regulation and expression patterns are free to diverge. Furthermore, enzymes that participate in a large number of alternative metabolic behaviors tend to be encoded by genes with a highly conserved sequence. Taken together, these findings support the hypothesis that maintaining large variability in the overall expression and metabolic repertoire of the cell is under marked evolutionary selection.

## Introduction

Recent comparative studies of genomic-scale gene expression levels have revealed substantial variation among different strains [1,2], among closely related species [3], and even within a genetically identical population [4–7]. Why do some of the genes monitored in these experiments manifest divergent expression values while the expression of others is constrained? We study this question from an evolutionary perspective in an in silico model of metabolism.

A key tool in studying metabolic networks is constraint-based modeling, which permits analysis of large-scale networks. Accurate prediction of dynamic metabolic activity requires kinetic models, but these rely on detailed information of the rates of enzyme activity, which is mostly unavailable, and are thus limited to small-scale networks. In contrast, constraint-based models use genome-scale networks to predict steady-state metabolic activity, regardless of specific enzyme kinetics. Stoichiometric, thermodynamic, flux capacity, and possibly other constraints are used to limit the space of possible flux distributions attainable by the metabolic network. Flux balance analysis (FBA) [8,9] is a specific, constraint-based method which assumes that the network is regulated to maximize or minimize a certain cellular function, which is usually taken to be the organism's growth rate. FBA has been successfully used for predicting growth, uptake rates, byproduct secretion, and growth following adaptive evolution, as well as other phenotypes [10–14].

The metabolic state predicted by FBA for a given growth media is not unique—in many cases there is a set of an infinite number of optimal solutions. Thus, we discuss here the *optimal solution space,* the space of all flux distributions leading to an optimal growth rate*.* On one hand, there are missing constraints in the model, and one line of research aims at reducing the solution space by adding biologically plausible constraints, e.g., by explicitly incorporating regulatory constraints in the model [15] and by looking for specific reactions for which new constraints may significantly reduce the size of the solution space [16]. On the other hand, even though there are still probably some missing constraints in FBA models, it has already been demonstrated that the solution space of such models does carry meaningful biological information [17,18]. Alternative optimal, steady-state flux solutions were shown to reflect redundant pathways [19], and sampling of the FBA solution space has been used, for example, to identify correlated fluxes in the mitochondrial metabolic network [18]. Moreover, Fong et al. [20,21] have shown that multiple biologically meaningful flux states are active in different conditions. However, this point has been largely ignored in many FBA studies which, focusing on a variety of other research questions, have examined an arbitrary single optimal solution.

Here we further pursue the possibility that the FBA solution space reflects multiple biologically meaningful metabolic states that are active in various conditions or under different evolutionary trajectories. We study the possibility that it reflects evolutionary constraints on expression and sequence of the associated genes. In particular, we focus on yeast metabolism using a recent genome-scale, fully compartmentalized model of Saccharomyces cerevisiae [22], which is one of the largest and most comprehensive metabolic models available for a microorganism. We show that by considering the entire FBA solution space we can identify constraints on the regulation of the associated genes, and thus predict which genes will have divergent expression levels among different yeast strains, and less conserved regulation among closely related species. In addition, we show that the sequences of genes that are active in multiple solutions tend to be highly conserved, suggesting that constraints on sequence divergence can also be discerned by studying the entire solution space. These results show that the space of FBA solutions, which emerges from a complex interplay between the stoichiometric constraints, the uptake rates defining the growth medium and the optimality assumption, is not just a technical consequence of our ignorance of additional constraints. Rather, this solution space sheds light on the evolution of metabolic regulation and of the metabolic network itself.

## Results

### Conservation of Expression Regulation Is Reflected by the FBA Solution Space

Cellular metabolism is governed by various factors such as enzyme kinetics, allosteric control, and transcriptional and post-transcriptional gene and protein regulation. Specifically, the effect of transcriptional regulation on cellular metabolism was previously studied based on gene expression measurements, small-scale flux measurements, and large-scale flux predictions. Enzyme-coding genes that form metabolic pathways were shown to be expressed just-in-time when needed in bacteria [23]. A strong qualitative correspondence between gene expression and metabolic fluxes for various pathways was shown in both bacteria and yeast, following environmental changes in yeast [24] and adaptive evolution in bacteria [25]. Previous studies have also shown that the expression patterns of enzyme-coding genes are correlated with the flux patterns predicted by FBA. Schuster et al. and Famili et al. have shown that genes associated with fluxes that are predicted to change together during a shift from one medium to another (e.g., in diauxic shift) are co-expressed under these conditions (but this was done on a small scale, especially the analysis of Schuster et al.), while Reed and Palsson have shown that the genes associated with fluxes that are correlated within the solution space also exhibit moderate levels of correlation in their expression [12,26,27].

Here we identify a more direct relationship between expression and flux. We compared mRNA transcript numbers [28,29] and protein levels [30] in rich media (yeast peptone dextrose [YPD]) with the predicted flux values when simulating YPD growth conditions (see Materials and Methods). As shown in Table 1, we find that the flux values show a moderate, statistically significant correlation with the corresponding gene expression levels and with protein abundance data measured via GFP fluorescence. Isozymes were not included in this analysis, but their inclusion yields qualitatively similar results (see Table 1 in Protocol S1).

**Table 1:**
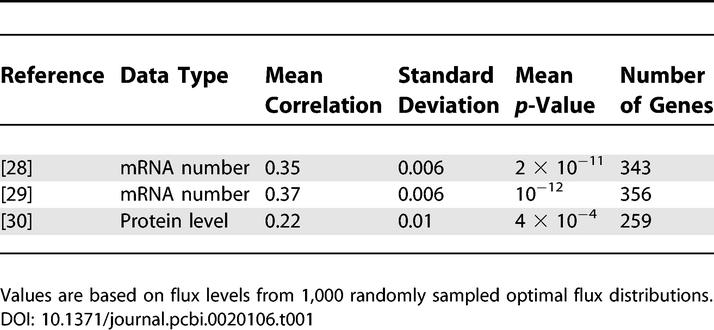
Spearman Rank Correlation between Flux Level and mRNA/Protein Abundance

**Figure 1:**
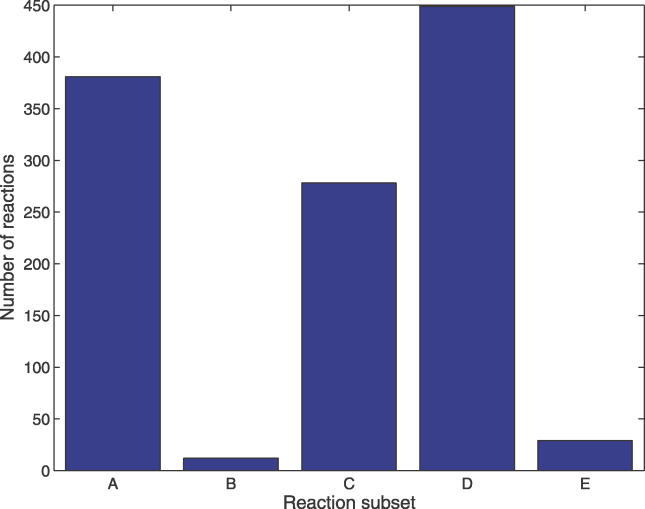
Distribution of the 1,149 Modelled Metabolic Reactions according to Their Variability within the Space of Optimal Solutions for YPD (A) Reactions with flux equal to zero in all optimal solutions. (B) Reactions with the same, nonzero value in all optimal solutions. (C) Reactions with low variability among optimal solutions *(flexbility score between 0 and 10^−2^)*. (D) Reactions with medium variability among optimal solutions *(flexbility score between 10^−2^ and 1/2)*. (E) Reactions with high variability among optimal solutions *(flexibility score at least 1/2)*.

Under most simulated conditions, FBA has an infinite number of optimal solutions. As shown in Figure 1, some reactions display a broad range of values within the set of optimal solutions for glucose-rich (YPD) conditions, while others have an almost fixed value in all optimal solutions. A reaction displays a broad range of values when there are alternative pathways to the one it belongs to. For example, when simulating glucose-rich conditions, the reaction along the glycolysis pathways that is catalyzed by Fba1, which converts fructose 1,6 bisphosphate into glyceraldehyde-3-phosphate and dihydroxyacetone phosphate, can have a flux approximately equal to that of glucose intake, or, alternatively, be bypassed completely via the pentose phosphate pathway.

As flux values are significantly correlated with expression levels, we hypothesized that the range of possible optimal flux values for a given reaction reflects evolutionary constraints on the expression levels of its associated enzymes. Specifically, the regulation of reactions that have an optimal fixed value is under strong selection to maintain their flux at the precise levels needed, while the regulation of reactions that may have a broad range of optimal values is under weaker selection.

To pursue this possibility we used *flux variability analysis* [19]: for each reaction we computed the maximal and minimal flux values attainable in the space of optimal flux distributions for growth conditions simulating YPD-rich media. We define the *YPD flexibility score* of a reaction as the difference between these maximal and minimal values. In addition, we performed a similar analysis, this time computing flux flexibility scores across 1,000 randomly generated growth media. In both cases, the flexibility score of a gene has been taken as the maximal score among the flexibility scores of the reactions it is associated with. Interestingly, we found that the YPD flexibility score is very similar to the flexibility scores found across the random media (mean Spearman rank correlation = 0.92), and focus on it in the sequel.

**Figure 2:**
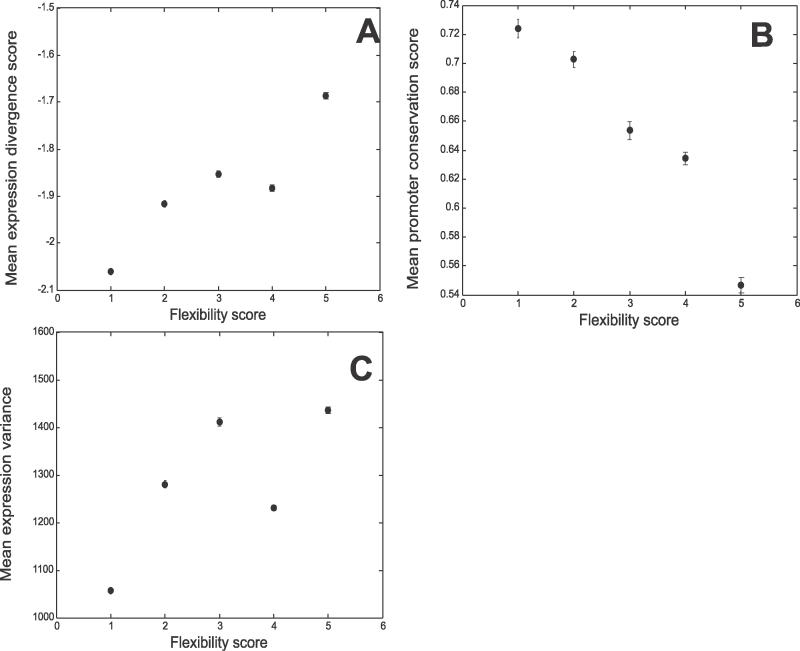
Correlation of YPD Flexibility Score with Biological Measurements Reflecting Constraints on Regulation (A) Mean expression divergence (from Townsend et al.) as a function of the gene's flexibility score; (B) Mean promoter conservation score (based on Harbison et al.) as a function of the gene's flexibility score; (C) Mean expression variance (from Ihmels et al.) as a function of the gene's flexibility score. Genes are binned according to their flexibility score into five bins, such that each bin contains the same number of genes. Plot-points represent mean *y*-axis values over these bins. Error bars depict mean standard error. Importantly, the correlations reported in the main text are computed from the raw data. The Spearman rank correlation of the binned data displayed here is 0.9 (A and C), and −1 (B).

To test the hypothesis that genes with smaller YPD flexibility scores have tighter regulation, we studied the correlation between the genes' YPD flexibility scores, the conservation of their promoters, and their expression patterns. We used a score computed by Townsend, Cavalieri, et al. [2], which measured expression patterns for four yeast strains, obtaining an *expression divergence score* for each gene. Harbison, Gordon, et al. [31] constructed a comprehensive map of S. cerevisiae transcription factor binding sites, and for each site reported the number of yeast species (from among *Saccharomyces paradoxus, Saccharomyces mikatae, and Saccharomyces bayanus*) in which it is conserved. Based on this data, we assigned to each gene a *promoter conservation score* (see Materials and Methods)*.* As shown in Figure 2A and 2B, both scores show moderate, statistically significant correlations with the YPD flexibility score; the Spearman rank correlation with expression divergence is 0.18 (*p*-value < 10^−4^, 469 genes), and is −0.18 with promoter conservation (*p*-value = 10^−3^, 330 genes). Although these statistically significant correlations are of moderate magnitude, taken together they support the hypothesis that low flexibility scores are associated with conserved regulation. We show that these results are robust with respect to the definition of the flexibility score and the analysis of near-optimal solution spaces in Table 2 in Protocol S2.

We also observed a statistically significant correlation when comparing the YPD flexibility score of a gene with its expression variability over a large set of conditions compiled by Ihmels, Bergmann, et al. ([32], only conditions relevant to YPD were considered—see Materials and Methods). As shown in Figure 2C, for the 600 genes analyzed, the Spearman rank correlation between these values is 0.17, with a *p*-value of 2 × 10^−5^. That is, their more flexible regulation does indeed manifest itself in increased expression variability.

A likely mechanism underlying these findings is that genes with high flexibility scores take part in pathways that have alternative ones for optimal growth. In some conditions one pathway is used, while in others the alternative is taken, leading to a variable expression pattern. This is reflected not only in the model, but also in comparison with lethality assays. Of the 50 genes with the highest flexibility scores, only 6% are essential for growth in YPD, whereas of the 89 that have a zero flexibility score, 17% are essential.

### Conservation of Gene Sequence Is Reflected by the FBA Solution Space

Papp et al. have shown that enzyme-coding genes that according to FBA are active in several conditions tend to be more conserved in evolution [33] and hence can be found in numerous organisms: they simulated nine representative growth media, and found an optimal flux distribution for each. They suggested that genes that are active (i.e., associated with reactions having nonzero flux) in only a few conditions are expected to be less conserved than their counterparts, since if these conditions become irrelevant, the gene may be lost. Indeed, they have shown that genes active in 0–7 conditions have, on average, fewer orthologs in other species than those that are active in eight or nine conditions.

We followed their analysis by carrying it out in a large-scale manner, and considered the entire optimal solution space rather than a single optimal solution. For each gene in the model we computed the fraction of 1,000 random growth media in which it is active using *flux variability analysis*. This value is the *intercondition activity score* of the reaction, and from it a similar score was deduced for the corresponding genes (see Materials and Methods). Rather than counting orthologs, as in [33], we compared this score directly with the evolutionary rate of the gene's sequence [34], and found a statistically significant anticorrelation between them, both in random rich media (Spearman rank correlation = −0.27; *p*-value = 2 × 10^−5^; 238 genes), and in random poor media (Spearman rank correlation = −0.35; *p*-value = 2 × 10^−8^; 238 genes).

**Figure 3:**
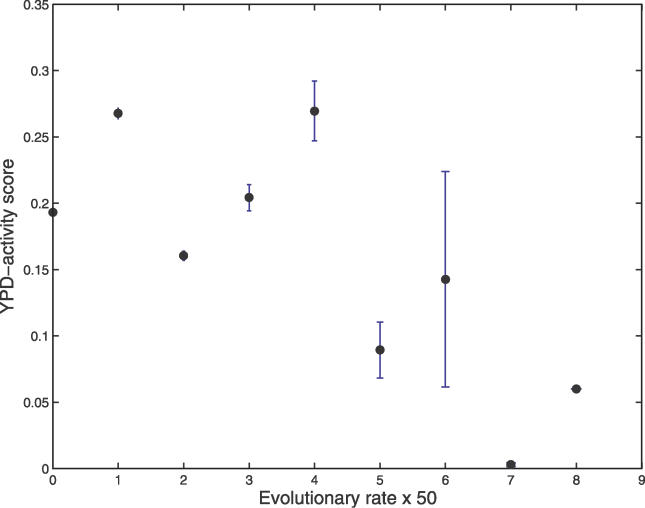
Average YPD Activity Score as a Function of a Gene's Evolutionary Rate Genes are binned by multiplying their evolutionary rate by 50, and rounding to the closest integer. The mean YPD activity score over each subset is depicted. Error bars describe mean standard deviation. Importantly, the correlations reported in the main text are computed from the raw data. The Spearman Rank correlation of the binned data displayed here is −0.71.

Surprisingly, sequence conservation can also be deduced from studying the solution space for a *specific* growth medium solely. To quantify the activity of yeast genes in rich media we computed for each gene a *YPD activity score,* reflecting the fraction of metabolic states in the near-optimal FBA solution space in which it is active (see Materials and Methods). Comparing this score to the evolutionary rate of the gene's sequence [34], we find a statistically significant anticorrelation between them (Figure 3; Spearman rank correlation = −0.37, *p*-value = 5 × 10^−9^, 234 genes), showing that the genes that are active at multiple metabolic states in a single growth media tend to be conserved. This correlation is also statistically significant when minimal–medium solutions are sampled, rather than those corresponding to YPD medium (Spearman rank correlation = −0.37, *p*-value = 3 × 10^−7^, 182 genes). One possible reason for this correlation is that it reflects the same trend observed already in multiple growth media: the metabolic network model captures stoichiometric-related constraints and nutrient limits, but may not include some environmental conditions. Hence, the FBA solution space for rich media may represent the various metabolic states that are optimal in rich media under different exogenous conditions, such as temperature or salinity. In other words, different metabolic states actually represent flux distributions under different conditions, and, as in the intercondition analysis, we expect that the sequence of genes that are required in many exogenous conditions will tend to be more conserved. Interestingly, the correlation observed in a single rich medium is higher than that obtained across multiple rich media, and remains statistically significant and qualitatively similar to that reported above when varying the near-optimality threshold of the solution space (Table 3 in Protocol S2).

Previously it was shown that protein dispensability, which is assessed using growth rate measurements following gene knockouts, is correlated with evolutionary sequence rate [34]. This correlation suggests that there is selection pressure to preserve the sequence of genes whose knockout has a notable effect on the growth rate of the organism. Repeating this analysis for the set of metabolic genes also revealed a statistically significant correlation between the knockout growth rate and evolutionary rate (Spearman rank correlation = 0.2, *p*-value = 0.04, 103 genes). However, notably, the latter correlation is weaker than that observed between the YPD activity score and evolutionary rate, further supporting the claim that the activation of genes in multiple conditions is a selected trait.

Previously it was shown that the best predictor for evolutionary rate is the expression level of the encoded gene [35]. Other plausible measures for gene importance, such as the number of physical interactions, essentiality, and the drop of fitness following a knockout, were reported to explain only a small fraction of the variation in expression rate [35,36]. Namely, their squared correlation coefficient between these measures and evolutionary rate are found to be 0.04–0.07, and the squared partial correlations, when controlling for expression level, are 0.02–0.06 [36]. By comparison, the YPD activity score attains an *r^2^*-value (squared rank correlation coefficient) of 0.17, and, importantly, remains significantly correlated with evolutionary rate even after the effect of mRNA expression level [28] is factored out (Pearson partial correlation is −0.25, and by linear regression the correlation with the residual has *r* ^2^ = 0.14; *p*-value = 1.6 × 10^−8^).

## Discussion

What do the metabolic flux distributions composing the FBA solution space represent? While some of them may be superfluous, arising from missing constraints, this study shows that as a whole, they are biologically meaningful. Three different possibilities for their interpretation suggest themselves: (i) effect of exogenous factors; (ii) alternative evolutionary pathways; and (iii) heterogeneity within a population.

### 

#### (i) Effect of Exogenous Factors.

In addition to the growth medium simulated by the model, the metabolic behavior of an organism is also influenced by exogenous factors that are beyond the model's scope. Hence, it is plausible that the stoichiometric constraints allow for the existence of a variety of different metabolic states that are needed for growth in the given medium under the various different external conditions that the organism may encounter. The solution space represents the union of these different conditions.

#### (ii) Alternative Evolutionary Pathways.

It was suggested that the FBA solution space may contain alternative flux distributions that are attainable through different evolutionary paths, as was experimentally shown in an adaptive evolution experiment in E. coli [19–21,25]. These experiments showed that although evolutionary endpoints may converge with respect to the selection pressure for high growth rate, the underlying metabolic states, characterized by measured metabolic fluxes and gene expression, may significantly diverge.

#### (iii) Heterogeneity within a Population.

Previous studies suggest that the predicted variability in metabolic states may represent heterogeneous metabolic behaviors of individuals within a cell population [17,19]. It is possible that the multiple metabolic states composing the space of optimal solutions represent this heterogeneity. This possibility is especially appealing in light of recent measurements of gene expression at the single-cell level, showing high variability in expression among cells taken from the same culture [4–7].

We find support for all three interpretations. The observation that reactions that display a range of values within the space of optimal solutions tend to be associated with genes whose regulation is less conserved supports interpretation (ii); where optimality allows for different evolutionary paths to be taken, and the data suggests that they are indeed taken. This finding is in agreement with the previous experiments of bacterial adaptive evolution, showing that adaptive mechanisms evolved in the transcriptional regulatory network that governs their metabolic state [20]. The correlation between the number of solutions in which a reaction is active and the conservation of the associated genes' sequence supports interpretation (i): The YPD activity score behaves similarly to the *inter*condition activity score, and thus may also be thought to reflect metabolic states across different (exogenous) conditions in YPD. But this finding can also be seen as supporting interpretation (ii)—enzymes that are active in multiple solutions, that is, according to interpretation (ii), appear in multiple strains, are more conserved. Indeed, as we find the YPD activity score to be a better predictor of sequence conservation than the intercondition activity score, it is likely that factors in addition in interpretation (i) play a role here.

Finally, the fact that reactions with variable flux values within the solution space tend to be associated with genes with variable expression patterns clearly supports interpretation (i), but can possibly also be seen as indirect support for interpretation (iii). While the expression variability we analyzed is based on averages over a population under different conditions, and interpretation (iii) addresses expression variability among individual cells in the same condition, it was suggested that these two measures of variability are related [37]. If this is indeed the case, then the results above indirectly support the possibility that variability within the solution space is correlated with variability within a population. However, additional large-scale measurements of population expression variance are required to establish this possibility further.

In fact, interpretations (i)–(iii) are not inclusive, and may correlate with different regions within the solution space. This suggests the following view of this space: some solutions are superfluous, and exist only due to the lack of sufficient regulatory (and other) constraints within the model; the remainder solution space is composed of subspaces representing solutions attained via different evolutionary paths; these spaces may be further partitioned to sets of solutions that arise under different external conditions, representing heterogeneous phenotypes among genetically similar cells.

In summary, we have shown that genes with a high potential flux range have indeed fewer constraints on their regulation, and that genes that are active in multiple metabolic states tend to be highly conserved. These results emerge when studying the FBA solution space as a whole, clearly showing that it carries meaningful biological information.

## Materials and Methods

### Metabolic network model.

The S. cerevisiae metabolic model used here is by [22]. It includes 1,149 reactions, associated with 734 genes. A reaction is considered active in a given flux distribution, if its associated flux is nonzero. Of the 1,149 reactions, 268 were identified to be essentially zero (absolute value at most 10^−10^) in all feasible (not necessarily optimal) flux distributions satisfying the stoichiometric constraints simulating YPD. Since these reactions are not relevant for growth in YPD, they were omitted when computing the YPD flexibility score and the YPD activity score.

In addition to the 1,149 internal reactions, we added to the model 116 uptake/excretion reactions, for each of the metabolites listed as extracellular in the basic model.

YPD growth conditions were simulated as in [13]. We list these reactions and their uptake rates in the Table S1.

The optimal solution space is defined as the set of all flux distributions that obey the stoichiometric and thermodynamic constraints, lead to a maximal growth rate, and minimize the sum of (absolute values of) the reactions. The latter constraint, following [38], aims to avoid futile flux cycles in the network which violate the laws of thermodynamics.

The entire analysis in this work was also applied to the metabolic model of [38], obtaining qualitatively similar results, and appears in Protocol S3.

### Datasets used.

The mRNA transcript numbers were taken from [28] and [29], which list values for 679 and 728 of the genes included in model, respectively.

Protein abundance, as measured through GFP fluorescence, was taken from [30,38,40], and was available for 475 of the encoding genes in the model.

Expression divergence measures were taken from the supplementary results of [2], 581 of which are for genes that appear in the model.

Expression variance values are based on the dataset compiled by [32], and were defined as the sum of squares of the log ratios (the same measure was used for this purpose in [37]).

Evolutionary rates were taken from [34], and were available for 379 of the genes in the model.

### Random sampling of the solution space.

The space of all flux distributions that obey the constraints imposed in FBA is a polyhedral set defined by the half-spaces corresponding to the constraints. Finding a flux distribution that maximizes the biomass reaction (which is a linear function of fluxes that produce essential biomass precursors) can thus be found using a simplex algorithm. Roughly, the algorithm travels from one vertex of the polyhedral set to a neighboring one, while improving the value of the objective function, until the function can no longer by improved (by convexity, this is indeed the global optimum). See, e.g., [41] for more details.

The rule by which the neighboring vertex is chosen when there are multiple possibilities is called a *pivot rule*. In a randomized simplex algorithm (cf. [41]), the pivot rule is simply to choose uniformly at random from among the vertices on which the objective function is improved. In the case where a whole set of optimal solutions exists, this leads to a solution that is chosen at random from among the optimal solutions.

We note that this is not a uniform sample of the solution space, since only *vertices* of the polyhedral set will be chosen and not internal points. But this is an appropriate sampling scheme when one is interested in sampling optimal flux distributions with extreme flux values since they correspond to vertices.

Indeed, the vertices of the polytope of optimal solutions are analogous to the much-studied extreme pathways [42] elementary flux modes [43,44]: the extreme pathways are the minimal set of flux distributions such that any feasible (not necessarily optimal) solution is a non-negative linear combination of them (i.e., they are the extreme rays of the feasible solutions cone); The vertices of the polytope of optimal solutions (which we sample) are the minimal subset of flux distributions such that any *optimal* solution is a non-negative linear combination of them.

### Simulating random growth media.

Random growth media were generated by setting limiting values to the uptake reactions independently at random. With probability *p,* the maximal uptake rate was set to 0—i.e., only excretion was allowed. Otherwise, uptake rate was limited to a value chosen uniformly at random between 0 and 1.

The values *p* = 0.5 and *p* = 0.95 were tested, simulating rich and poor media, respectively. Eight of the uptake rates were taken positive in all media, to ensure viability (for water, ammonium, phosphate, sulfate, oxygen, sodium, potassium, and carbon dioxide).

A similar sampling method was used in [45].

### Comparing flux level with mRNA/protein expression level.

Comparing flux level with mRNA level and protein level requires inferring a “flux level” for a gene, based on the reactions it is associated with. For each gene, we defined this to be the maximal flux level predicted for its associated reactions, as this is the level that most constraints it.

Analysis of isozymes is deferred to Protocol S1, where the correlation between the two measures is based on reactions rather than on genes.

In Protocol S2's Table 4 we also show that the correlation between flux level and expression level is statistically significant when near-optimal solution spaces are considered.

### Gene flexibility score.

The flexibility score of a reaction in a given medium is the difference between the maximal flux that can flow through it in an optimal flux distribution, and the minimal one. This is computed using flux variability analysis [19].

The flexibility score of a gene in a given medium is the maximum of the flexibility scores for the reactions it is associated with. The rationale for using the maximum value is that this value is the one that most constrains the required enzyme quantity for obtaining optimal flux. That is, if a protein is associated with several reactions, for its expression level to comply with all optimal flux values, it most complies with the highest one.

For isozymes, the definition is slightly more complicated. Suppose a reaction *R* can attain values between *x* and *y,* and hence its flexibility score is *y* minus *x*. If a single gene is associated with this reaction, then, as defined above, the reaction's contribution towards the gene's flexibility score is simply *y* minus *x*, since we think of this range of fluxes as defining the flexibility of the gene's expression. However, if there are several isozymes associated with the reaction, then their flexibility can, potentially, be larger.

If *x >* 0, then the values an associated isozyme *I* can attain are from 0 to *y,* since if the expression level of *I* is smaller than *x,* the other isozymes can compensate for it, putting the total reaction rate with the optimal range of [x,y]. Hence, in this case the contribution of the reaction towards the gene's flexibility is *y*, rather than *y* minus *x*. Similarly, if *y* < 0, an isozyme could attain any value in the range [x,0], and hence the contribution of the reaction towards the gene's flexibility would be *|x|*. Taken together, we define contribution of a reaction attaining flux values in the range [x,y] towards the flexibility score of its associated isozymes as the maximum among *y* minus *x, y,* and *|x|*.

### Promoter conservation score.

Transcription factors' binding sites were considered as regulating a gene if they appear in the map of [31] within 500 bp of the gene's translation start site.

Each binding site received a score of 1, if it is conserved in all three species from among *S. paradoxus, S. mikatae, and S. bayanus;* a score of 0.5 if it conserved in two of them; and a score of 0 otherwise. The *promoter conservation score* for a gene is the mean score for the binding sites associated with it.

Data was available for 382 genes.

### Gene medium-specific activity score.

The activity score of a gene in a given medium is obtained by sampling the solution space, and by counting the number of solutions that have reactions associated with it that are active.

When doing so by sampling the space of optimal solutions for YPD, we got an activity score of either 0 or 1 for 90% of the genes. To obtain a range of activity scores that can be better differentiated between activity scores we sampled the space of near-optimal solutions, as was done in [26,45]. Specifically, we sampled the space of solutions with growth rate at least 80% of the optimal one in YPD using the random simplex algorithm. In Protocol S2's Table 3 we show that similar results are obtained when the 80% threshold value is varied to sample other near-optimal solution spaces.

Genes that encode enzymes which have isozymes were excluded from the analysis, since their knockout has no effect on the network. In Protocol S1 we analyze possible inclusions of isozymes in this analysis.

Genes that are active in all solutions are also excluded, since we expect many of them to be essential genes, and it is known that essential genes tend to have conserved sequence. If they are included, the Spearman rank correlation between the YPD activity score and sequence divergence is indeed more distinct: r = −0.41, *p* = 1.3 × 10^−12^, 270 genes.

### Gene intercondition activity score.

The intercondition activity score of a gene was computed from 1,000 random growth media (see the section, Simulating random growth media, above), and computed similarly to the medium-specific activity score: For each gene, its interactivity score is the number of random media in which it is active, where a gene is considered active if one the reactions for which the gene is required is active.

As for the medium-specific activity score, we omitted from the analysis genes that are active in all conditions. If these genes are included, the Spearman rank correlation between the intercondition activity score and sequence divergence is more distinct: r = −0.31 for rich growth conditions, and r = −0.37 for poor growth conditions.

## Supporting Information

Dataset S1 IDs of Genes Included in the Analysis (5 KB TXT)

Protocol S1 Considering the Effect of Isozymes (58 KB DOC)

Protocol S2 Robustness Analysis (73 KB DOC)

Protocol S3 Consistency of the Results with the Model of Kuepfer et al. (70 KB DOC)

Table S1 Rate of Uptake Reactions Modelling YPD Medium (136 KB DOC)

### Accession Numbers

For the accession numbers of the yeast genes analyzed in this paper, from the Saccharomyces Genome Database (http://www.yeastgenome.org), see Dataset S1.

## Abbreviations

FBA: flux balance analysis
YPD: yeast peptone dextrose
