# Supplementary material for: Conservation of Expression and Sequence of Metabolic Genes Is Reflected by Activity Across Metabolic States
Source: PLoS Comput Biol. 2006 Aug 18;2(8):e106. doi: 10.1371/journal.pcbi.0020106 (PMC1550272; doi:10.1371/journal.pcbi.0020106)
Supplement: Supplementary file 1 [file pcbi.0020106.sd002.doc]

**Supplementary Results 1 : Considering the effect of isozymes in our analysis**.

An inherent problem in Flux Balance Analysis is the role of isozymes – a number of enzymes which can, independently, catalyze the same reaction. Isozymes for the same reaction are often under different regulation, and hence have different expression patterns. However, in FBA we can not differentiate between them, as we analyze the fluxes of reactions, rather than enzyme expression levels. Moreover, in the model of Duarte et al.[1], 215 of the reactions (20%) are associated with isozymes, so their effect can not be assumed to be negligible.

In our analysis, the effect of isozymes enters in three places: (i) In analyzing the correlation between flux level and mRNA/protein level; (ii) In defining the flexibility score of a gene; and (iii) In defining the activity score of a gene.

**1.1 Correlation between metabolic flux level and mRNA/protein level**

To include isozymes in the analysis of the correlation between flux level and mRNA/protein level, we computed the correlation over reactions rather than genes.

For each reaction which is catalyzed by several isozymes, we think of the reaction as being catalyzed by the total concentration (expression level) of all relevant isozymes.

Hence, we compare the reaction's predicted flux level to the total amount of relevant isozyme expression levels. If an enzyme is composed of a protein complex, we take its expression level to be the minimal expression level among the proteins that form the complex.

Formally, suppose the expression level of *gene_i1_* is *g_i1_*, that of *gene_i2_* is *g_i2_*, and so on. For a reaction that is listed as being catalyzed by:

*(gene_i1_ and gene_i2_ and gene_i3_ …) or (gene_j1_ and gene_j2_ and gene_j3_ …) or …*,

we compare its predicted flux value to:

*min(g_i1_, g_i2_, g_i3_ …) + min(g_j1_, g_j2_, g_j3_ …) + …*

Supplementary Table 1 lists the mean correlations obtained in this way.

| **Source** | **Data type** | **Mean correlation** | **Standard Deviation** | **Mean p-value** | **Number of reactions** |
| --- | --- | --- | --- | --- | --- |
| [2] | mRNA number | 0.22 | 0.005 | 4.5x10^-8^ | 599 |
| [3] | mRNA number | 0.23 | 0.005 | 7.2x10^-9^ | 644 |
| [4] | Protein level | 0.11 | 0.007 | 0.02 | 465 |

**Supplementary Table 1:** Spearman rank correlation between flux level and mRNA/protein abundance, when isozymes are included. Values are based on flux levels from 1000 randomly sampled optimal flux distributions.

**1.2 Gene flexibility score**

As described in the main text, the flexibility score of an isozyme can be computed in a fairly straightforward manner – we allow for greater flexibility, by considering the isozyme as corresponding to a flux range form 0 (not active) to the maximal reaction flux (when it is the sole enzyme catalyzing the reaction).

**1.3 Gene YPD-activity score**

The activity score represents the expected number of solutions in which a gene is active. For an active reaction associated with several isozymes, we think of each isozyme as having equal probability of being active and catalyzing the reaction. That is, we assume that isozymes are not concurrently active [8].

Hence, extending the activity score to isozymes, when a reaction associated with *k* isozymes is active in a sampled solution, we add *1/k* to the expected number of solutions in which each of the isozymes is active. This is equivalent to designating one of them as active uniformly at random.

The Spearman rank correlation between the activity score defined in this way, and sequence divergence ([5]) is -0.35, corresponding to a p-value of 1.4x10^-11^ (343 genes). This is similar to the correlation obtained without isozymes (r=-0.37) over a smaller set of 234 genes.

**References**

1. Duarte NC, Herrgard MJ, Palsson BO (2004) Reconstruction and validation of Saccharomyces cerevisiae iND750, a fully compartmentalized genome-scale metabolic model. Genome Res 14: 1298-1309.

2. Holstege FC, Jennings EG, Wyrick JJ, Lee TI, Hengartner CJ, et al. (1998) Dissecting the regulatory circuitry of a eukaryotic genome. Cell 95: 717-728.

3. Velculescu VE, Zhang L, Zhou W, Vogelstein J, Basrai MA, et al. (1997) Characterization of the yeast transcriptome. Cell 88: 243-251.

4. Huh WK, Falvo JV, Gerke LC, Carroll AS, Howson RW, et al. (2003) Global analysis of protein localization in budding yeast. Nature 425: 686-691.

5. Hirsh AE, Fraser HB (2001) Protein dispensability and rate of evolution. Nature 411: 1046-1049.

6. Townsend JP, Cavalieri D, Hartl DL (2003) Population genetic variation in genome-wide gene expression. Mol Biol Evol 20: 955-963.

7. Harbison CT, Gordon DB, Lee TI, Rinaldi NJ, Macisaac KD, et al. (2004) Transcriptional regulatory code of a eukaryotic genome. Nature 431: 99-104.

8. Ihmels J, Bergmann S, Barkai N (2004) Defining transcription modules using large-scale gene expression data. Bioinformatics 20: 1993-2003.

9. Kuepfer L, Sauer U, Blank LM (2005) Metabolic functions of duplicate genes in Saccharomyces cerevisiae. Genome Res 15: 1421-1430.
