# Supplementary material for: Conservation of Expression and Sequence of Metabolic Genes Is Reflected by Activity Across Metabolic States
Source: PLoS Comput Biol. 2006 Aug 18;2(8):e106. doi: 10.1371/journal.pcbi.0020106 (PMC1550272; doi:10.1371/journal.pcbi.0020106)
Supplement: Supplementary file 2 [file pcbi.0020106.sd003.doc]

**Supplementary Results 2 : Robustness analysis**

**2.1 Robustness of flexibility score correlations**

Recall that the flexibility score of a reaction is based on its extreme values within the space of optimal growth flux distributions. Here we examine the robustness of the correlations associated with this score, by varying two parameters in its definition:

1. Looking at solution spaces of near-optimal growth flux distributions, with growth rates above a given threshold.
2. Defining the flexibility score of a gene as the average flexibility of its associated reactions, rather than the maximal value.

We report the correlation of the score with expression divergence ([1]), promoter conservation ([2]), and expression variability ([3]), denoted by ED, PC and EV correlation, respectively, in Supplementary Table 2. Notably, the correlations are robust to these alternative definitions.

| **Fraction of optimality** | **Gene-reaction association** | **ED correlation** | **PC correlation** | **EV correlation** |
| --- | --- | --- | --- | --- |
| 1 | Max | 0.18 ; 10^-4^ | -0.18 ; 10^-3^ | 0.17 ; 2x10^-5^ |
| 0.95 | Max | 0.23 ; 8x10^-7^ | -0.18 ; 10^-3^ | 0.18 ; 5x10^-5^ |
| 0.9 | Max | 0.2 ; 10^-5^ | -0.15 ; 5x10^-3^ | 0.15 ; 10^-4^ |
| 1 | Average | 0.18 ; 10^-4^ | -0.19 ; 4x10^-4^ | 0.17 ; 2x10^-5^ |
| 0.9 | Average | 0.22 ; 10^-6^ | -0.15 ; 6x10^-3^ | 0.18 ; 10^-5^ |
| 0.95 | Average | 0.22 ; 2x10^-6^ | -0.15 ; 4x10^-3^ | 0.16 ; 7x10^-5^ |

**Supplementary Table 2:** Spearman rank correlation of flexibility score with three measures of variation in regulation, based on varying definitions of this score. Fraction of optimality denotes the constraint on the growth rate w.r.t. the maximal growth rate, defining the space of near-optimal solutions considered. Gene-reaction association lists whether a gene's score is the maximal among associated reactions or the average one. Entries in the right three columns are the Spearman rank correlations and the corresponding p-values.

**2.2 Robustness of YPD-activity score correlations**

Supplementary Table 3 examines the robustness of the correlation between activity score and sequence evolutionary rate, looking at solution spaces of near-optimal growth flux distributions. We note that for the space of strictly optimal solutions, we do not get a significant correlation with evolutionary rate. This is because for the 270 genes for which data is available, 69 get a score of 1 (and were omitted, to avoid biasing the results, as noted in the main text), and 175 get a score of 0, leaving only 26 genes with a non-trivial score. If genes with score 1 are not omitted, the correlation coefficient is -0.3 (p-value = 6.5x10^-7^).

| **Fraction of optimality** | **Spearman rank correlation with evolutionary rate** | **p-value** | **Number of genes** |
| --- | --- | --- | --- |
| 1 | -0.064 | Not significant | 201 |
| 0.9 | -0.373 | 3.7x10^-9^ | 234 |
| 0.8 | -0.370 | 5.4x10^-9^ | 234 |
| 0.7 | -0.370 | 5x10^-9^ | 234 |

**Supplementary Table 3:** Spearman rank correlation of YPD-activity score with sequence evolutionary rate. Fraction of optimality denotes the solution space analyzed - the value listed as the fraction of the optimal growth rate is the threshold on the growth rate which defines the space of near-optimal solutions considered.

**2.3 Robustness of the flux level – expression level correlation**

Supplementary Table 4 examines the correlation between flux level and that of mRNA and protein, where the flux level is sampled from near-optimal solution spaces.

| **Source** | **Data type** | **Mean correlation**  **at 1.0 FOO** | **Mean correlation**  **at 0.9 FOO** | **Mean correlation**  **at 0.8 FOO** | **Mean correlation**  **at 0.7 FOO** |
| --- | --- | --- | --- | --- | --- |
| [4] | mRNA number | 0.35 | 0.37 | 0.35 | 0.35 |
| [5] | mRNA number | 0.37 | 0.35 | 0.33 | 0.33 |
| [6] | Protein level | 0.22 | 0.24 | 0.23 | 0.23 |

**Supplementary Table 4:** Spearman rank correlation of flux level and mRNA/protein level, across various near-optimal solution spaces. The four right columns list mean correlation over varying levels of the fraction of optimality (FOO) threshold for the growth rate.

**References**

1. Townsend JP, Cavalieri D, Hartl DL (2003) Population genetic variation in genome-wide gene expression. Mol Biol Evol 20: 955-963.

2. Harbison CT, Gordon DB, Lee TI, Rinaldi NJ, Macisaac KD, et al. (2004) Transcriptional regulatory code of a eukaryotic genome. Nature 431: 99-104.

3. Ihmels J, Bergmann S, Barkai N (2004) Defining transcription modules using large-scale gene expression data. Bioinformatics 20: 1993-2003.

4. Holstege FC, Jennings EG, Wyrick JJ, Lee TI, Hengartner CJ, et al. (1998) Dissecting the regulatory circuitry of a eukaryotic genome. Cell 95: 717-728.

5. Velculescu VE, Zhang L, Zhou W, Vogelstein J, Basrai MA, et al. (1997) Characterization of the yeast transcriptome. Cell 88: 243-251.

6. Huh WK, Falvo JV, Gerke LC, Carroll AS, Howson RW, et al. (2003) Global analysis of protein localization in budding yeast. Nature 425: 686-691.

7. Duarte NC, Herrgard MJ, Palsson BO (2004) Reconstruction and validation of Saccharomyces cerevisiae iND750, a fully compartmentalized genome-scale metabolic model. Genome Res 14: 1298-1309.

8. Kuepfer L, Sauer U, Blank LM (2005) Metabolic functions of duplicate genes in Saccharomyces cerevisiae. Genome Res 15: 1421-1430.
