# Supplementary material for: Conservation of Expression and Sequence of Metabolic Genes Is Reflected by Activity Across Metabolic States
Source: PLoS Comput Biol. 2006 Aug 18;2(8):e106. doi: 10.1371/journal.pcbi.0020106 (PMC1550272; doi:10.1371/journal.pcbi.0020106)
Supplement: Supplementary file 3 [file pcbi.0020106.sd004.doc]

**Supplementary Results 3 : Consistency of the results with the model of Kuepfer et al.**

The analysis presented in this work is based on the metabolic model of Duarte et al. [1]. An alternative model was constructed by Kuepfer et al. [2], which, while being somewhat less comprehensive, is reported to yield a better prediction of gene lethality.

In Supplementary Tables 5-7 we describe the results of our analysis when applied to the model of Kuepfer et al. In Supplementary Table 5 we report the correlation between flux level and mRNA/protein level. In Supplementary Table 6 we report the correlation between flexibility score and the aforementioned measures, for varying levels of fraction of optimality (as in the previous section). In Supplementary Table 7 we report the correlation between YPD-activity score and sequence divergence.

| **Source** | **Mean correlation** | **Standard Deviation** | **Mean p-value** | **Number of genes** |
| --- | --- | --- | --- | --- |
| [3] | 0.34 | 0.007 | 1.6x10^-10^ | 325 |
| [4] | 0.35 | 0.008 | 4x10^-11^ | 345 |
| [5] | 0.21 | 0.007 | 7x10^-4^ | 251 |

**Supplementary Table 5:** Spearman rank correlation between flux level in the Kuepfer model, and mRNA (Source [3], [4]) and protein level (Source [5]). Results are based on 1000 random optimal flux distributions.

| **Fraction of optimality** | **ED correlation** | **PC correlation** | **EV correlation** |
| --- | --- | --- | --- |
| 1 | 0.12 ; 0.01 | -0.14 ; 0.01 | 0.12 ; 0.003 |
| 0.95 | 0.17 ; 10^-4^ | -0.15 ; 9x10^-3^ | 0.16 ; 8x10^-5^ |
| 0.9 | 0.17 ; 2x10^-4^ | -0.1 ; 0.05 | 0.14 ; 3x10^-4^ |

**Supplementary Table 6:** Spearman rank correlation of flexibility score, based on optimal and near-optimal solution spaces for the metabolic model of Kuepfer et al. Fraction of optimality denotes the constraint on the growth rate w.r.t. the maximal growth rate. The three columns to the right are as in Supplementary Table 2.

| **Spearman rank correlation** | **p-value** | **Number of genes** |
| --- | --- | --- |
| -0.23 | 0.004 | 152 |

**Supplementary Table 7:** Spearman rank correlation between gene YPD-activity score based on the metabolic model of Kuepfer et al., and sequence divergence.

**References**

1. Duarte NC, Herrgard MJ, Palsson BO (2004) Reconstruction and validation of Saccharomyces cerevisiae iND750, a fully compartmentalized genome-scale metabolic model. Genome Res 14: 1298-1309.

2. Kuepfer L, Sauer U, Blank LM (2005) Metabolic functions of duplicate genes in Saccharomyces cerevisiae. Genome Res 15: 1421-1430.

3. Holstege FC, Jennings EG, Wyrick JJ, Lee TI, Hengartner CJ, et al. (1998) Dissecting the regulatory circuitry of a eukaryotic genome. Cell 95: 717-728.

4. Velculescu VE, Zhang L, Zhou W, Vogelstein J, Basrai MA, et al. (1997) Characterization of the yeast transcriptome. Cell 88: 243-251.

5. Huh WK, Falvo JV, Gerke LC, Carroll AS, Howson RW, et al. (2003) Global analysis of protein localization in budding yeast. Nature 425: 686-691.
