# Supplementary material for: Conservation of Expression and Sequence of Metabolic Genes Is Reflected by Activity Across Metabolic States
Source: PLoS Comput Biol. 2006 Aug 18;2(8):e106. doi: 10.1371/journal.pcbi.0020106 (PMC1550272; doi:10.1371/journal.pcbi.0020106)
Supplement: Supplementary file 4 [file pcbi.0020106.st001.doc]

**Supplementary Data : Uptake reactions**

The following reactions were added to the iND750 metabolic model [1], with the listed limit on their rate. Metabolite abbreviations are as per the iND750 model.

| **Metabolite** | **Uptake rate** |
| --- | --- |
| h2o[e] | Unbound |
| sucr[e] | 0 (Excreted) |
| fru[e] | 0 (Excreted) |
| glc-D[e] | 20 |
| asn-L[e] | 0.5 |
| asp-L[e] | 0.5 |
| nh4[e] | Unbound |
| fmn[e] | 0 (Excreted) |
| pi[e] | Unbound |
| ribflv[e] | 0 (Excreted) |
| 13BDglcn[e] | 0 (Excreted) |
| thmmp[e] | 0 (Excreted) |
| thm[e] | 0 (Excreted) |
| thmpp[e] | 0 (Excreted) |
| h[e] | 0 (Excreted) |
| zymst[e] | 0 (Excreted) |
| xyl-D[e] | 0 (Excreted) |
| xylt[e] | 0 (Excreted) |
| xtsn[e] | 0 (Excreted) |
| xan[e] | 0 (Excreted) |
| val-L[e] | 0.5 |
| uri[e] | 0 (Excreted) |
| urea[e] | 0 (Excreted) |
| ura[e] | 0 (Excreted) |
| tyr-L[e] | 0.5 |
| ttdca[e] | 0 (Excreted) |
| trp-L[e] | 0.5 |
| tre[e] | 0 (Excreted) |
| thym[e] | 0 (Excreted) |
| thr-L[e] | 0.5 |
| thymd[e] | 0.5 |
| succ[e] | 0 (Excreted) |
| srb-L[e] | 0 (Excreted) |
| sprm[e] | 0 (Excreted) |
| spmd[e] | 0 (Excreted) |
| so4[e] | Unbound |
| ser-L[e] | 0.5 |
| sbt-L[e] | 0 (Excreted) |
| sbt-D[e] | 0 (Excreted) |
| rib-D[e] | 0 (Excreted) |
| pyr[e] | 0 (Excreted) |
| ptrc[e] | 0 (Excreted) |
| pro-L[e] | 0.5 |
| pnto-R[e] | 0 (Excreted) |
| phe-L[e] | 0.5 |
| pepd[e] | 0 (Excreted) |
| pap[e] | 0 (Excreted) |
| orn[e] | 0 (Excreted) |
| ocdcya[e] | 0 (Excreted) |
| ocdcea[e] | 0 (Excreted) |
| ocdca[e] | 0 (Excreted) |
| o2[e] | 2 |
| nmn[e] | 0 (Excreted) |
| na1[e] | Unbound |
| mmet[e] | 0 (Excreted) |
| met-L[e] | 0.5 |
| melib[e] | 0 (Excreted) |
| man[e] | 0 (Excreted) |
| mal-L[e] | 0 (Excreted) |
| malt[e] | 0 (Excreted) |
| lys-L[e] | 0.5 |
| leu-L[e] | 0.5 |
| lac-L[e] | 0 (Excreted) |
| k[e] | Unbound |
| ins[e] | 0 (Excreted) |
| inost[e] | 0 (Excreted) |
| ile-L[e] | 0.5 |
| hxan[e] | 0 (Excreted) |
| his-L[e] | 0.5 |
| hdcea[e] | 0 (Excreted) |
| hdca[e] | 0 (Excreted) |
| gua[e] | 0.5 |
| gthox[e] | 0 (Excreted) |
| gsn[e] | 0 (Excreted) |
| gly[e] | 0.5 |
| glyc[e] | 0 (Excreted) |
| glu-L[e] | 0 (Excreted) |
| gln-L[e] | 0.5 |
| gcald[e] | 0 (Excreted) |
| gam6p[e] | 0 (Excreted) |
| gal[e] | 0 (Excreted) |
| fum[e] | 0 (Excreted) |
| for[e] | 0 (Excreted) |
| etoh[e] | 0 (Excreted) |
| ergst[e] | 0 (Excreted) |
| duri[e] | 0 (Excreted) |
| dttp[e] | 0 (Excreted) |
| din[e] | 0 (Excreted) |
| dgsn[e] | 0 (Excreted) |
| dcyt[e] | 0.5 |
| dann[e] | 0 (Excreted) |
| dad-2[e] | 0 (Excreted) |
| cytd[e] | 0 (Excreted) |
| cys-L[e] | 0.5 |
| csn[e] | 0 (Excreted) |
| crn[e] | 0 (Excreted) |
| co2[e] | Unbound |
| cit[e] | 0 (Excreted) |
| chol[e] | 0 (Excreted) |
| btn[e] | 0 (Excreted) |
| arg-L[e] | 0.5 |
| arab-L[e] | 0 (Excreted) |
| arab-D[e] | 0 (Excreted) |
| amet[e] | 0 (Excreted) |
| alltt[e] | 0 (Excreted) |
| alltn[e] | 0 (Excreted) |
| ala-L[e] | 0.5 |
| akg[e] | 0 (Excreted) |
| adn[e] | 0 (Excreted) |
| ade[e] | 0.5 |
| ac[e] | 0 (Excreted) |
| acald[e] | 0 (Excreted) |
| 4abut[e] | 0 (Excreted) |
| abt[e] | 0 (Excreted) |
| 8aonn[e] | 0 (Excreted) |
| 5aop[e] | 0 (Excreted) |

**References**

1. Duarte NC, Herrgard MJ, Palsson BO (2004) Reconstruction and validation of Saccharomyces cerevisiae iND750, a fully compartmentalized genome-scale metabolic model. Genome Res 14: 1298-1309.
